# A Prospective, Randomized, Double-Blind Study on the Efficacy of Different Modes of Topical Application of Nasal Anesthetics in the Diagnostic Nasal Endoscopy Procedure

**DOI:** 10.7759/cureus.29436

**Published:** 2022-09-21

**Authors:** Prabu Velayutham, Prem Davis, Surya Ravichandran, Joemol John

**Affiliations:** 1 Department of Ear Nose Throat, Sri Venkateshwaraa Medical College Hospital and Research Centre, Puducherry, IND

**Keywords:** pain, cotton pledgets, nasal packing, lignocaine, sprays

## Abstract

Introduction

In the current otorhinolaryngology practice, technology has always been an essential part. Therefore, diagnostic nasal endoscopy (DNE) has become a vital examination in today’s practice. In order to visualize the nasal cavity in a systematic manner without any discomfort to both patient and doctor, the nose should be well anesthetized and decongested.

Objective

The study is to compare and evaluate the efficacy of 4% lignocaine-oxymetazoline cotton pledget packing versus topical sprays in the preparation of nasal cavities for DNE.

Methodology

The prospective, randomized, double-blind study was conducted among 246 patients and was divided into two groups. In the first group, the nose was packed with cotton pledgets containing 4% lignocaine-oxymetazoline and another group with 4% lignocaine-oxymetazoline spray. Following DNE, patients and surgeons were questioned on a pre-formed questionnaire to evaluate their experience during the procedure.

Results

It was observed that the time taken for the pre-endoscopic preparation of the packing group was more than the spray group. A total of 91.9% of the spray group had pain during the pre-endoscopic preparation and more burning and tingling sensation than in the nasal pack (75.6%). A total of 69.9% of the patients among the spray group participants compared to 32.5% of the packing group patients experienced more throat discomfort. In addition, 12% of the packing group had mucosal bleeding during the preparation. A total of 32.5% of the spray group experienced severe pain when compared to 12.2% of the packing group during the endoscopic procedure. Most of the participants from both groups had difficulty visualizing the superior turbinate and sphenoethmoidal recess during the procedure. There was a significant difference seen between both the groups with respect to pain during the pre-endoscopic procedure (p=0.0005), burning/tingling sensation (p<0.0001), throat pain (<0.0001), mucosal bleed (p=0.0003), pain during the procedure (p=0.0001), and discomfort after the procedure (p<0.0001).

Conclusion

Both methods of nasal preparation have merits and demerits in terms of discomfort, pain, and visualization of structures. Still, the packing of the nasal cavity with cotton pledgets is better when compared to spraying with 4% lignocaine-oxymetazoline. However, 4% lignocaine-oxymetazoline spray can be used during an emergency situation and with sensitive patients.

## Introduction

In the current otorhinolaryngology practice, technology has always been an essential part, and the advent of diagnostic nasal endoscopy (DNE) is proof of this [1]. DNE is becoming one of the most widespread and valuable examinations in the otorhinolaryngology practice today [2]. For the evaluation of the diseases of nasal and paranasal sinuses ranging from the deviated nasal septum to malignant conditions, DNE is the procedure of choice [3]. Before the discovery of the endoscopes, it was very challenging to visualize the normal and hidden structures of the nose and paranasal sinuses. However, Hopkins's development of optic rod endoscopes in 1960 has revolutionized the optical quality available to surgeons [4]. This discovery has made a revolution in the surgical management of nasal and paranasal sinus diseases from radical to minimally invasive procedures [4, 5]. They allow detailed examination of the hidden areas of the nose, which are not possible by the conventional way of using a standard head mirror examination [6]. In addition, DNE is considered more sensitive than a CT scan in accessing diseases in certain nose areas [3]. To visualize the nasal cavity systemically without causing any discomfort to the patient and doctor, the nasal mucosa should be topically anesthetized and decongested before the procedure. Though this procedure is very common, the preparation of the nose by topical anesthetizing and decongestant agents has not been standardized till now. Most patients experience discomfort during the process of preparing the nose with a topical anesthetizing agent. So, the critical aspect is to use a topical agent of an optimal dose without causing any discomfort to the patient and providing a good field of vision during the endoscopic procedure. Packing the nasal cavities with cotton pledgets soaked with lignocaine and decongestant has been used for a long time, even though it causes discomfort to the patient [7]. On the other hand, the technique of using a topical spray is also found to be effective with minimal discomfort to the patients without compromising the ease of visualization to the surgeon [8]. This prospective, randomized, double-blind study aims to compare the efficacy of 4% lignocaine-oxymetazoline cotton pledgets with 4% lignocaine-oxymetazoline spray in local anesthetic, evaluate the decongestant action on the nasal mucosa in the preparation of DNE and patient's acceptance and doctors ease of performing the procedure.

## Materials and methods

This prospective, randomized, double-blind study was conducted among patients who were more than 18 years of age visiting our institution's outpatient department from September 2021 to February 2022 with an indication for the DNE included in the study. Pregnant women, patients with a history of nasal surgery, tumors of the nose and paranasal sinuses, and patients with uncontrolled hypertension were excluded from the study.

Sampling

Mishra P et al. [9] found that the absence of pain during the procedure based on the visual analog scale was 16% and 4% among group A and group B, respectively, with an alpha error of 5% and power of 80%. Therefore, the minimum sample size required per group was 114, calculated using the software OpenEpi for hypothesis testing between two proportions formula. So, the total minimum sample size is 228. Considering a non-response rate of 10%, an additional 10% has been added to the sample size. So, the total sample size is 246.

Interventions

After the initial assessment, 246 patients were categorized into groups A and B equally by systematic random sampling. The patients in Group A underwent nasal packing on both sides with cotton pledgets admixed with 9 ml of 4% lignocaine with 1 ml of oxymetazoline 0.05% w/v. Three cotton pledgets had been placed in the nasal cavity, one on the floor of the nasal cavity, the second pledget in the medial to the middle turbinate, and the third pledget in the middle meatus [10]. The patients in group B underwent two puffs of the nasal spray of oxymetazoline (each ml contains 0.5 mg, each puff contains 0.05 mg. So, two puffs contained 0.1 mg), followed by two puffs of 4% lignocaine spray after one minute in both nostrils by placing the end of the spray at the vestibule of the nose. Patients of both groups had been made to wait for 10 minutes for the anesthetic to act. Shortly after anesthetizing the nasal mucosa, the patient was asked about the level of discomfort (based on the visual analogue scale of 1 to 10) [11]. The time taken to do the nasal packing and spraying was noted. The patient was enquired about the burning/tingling sensation in the throat and the development of throat discomfort during the waiting period. Then DNE was performed with a 0-degree rigid endoscope. The procedure consisted of three passes, the first pass to visualize the floor, septum, nasopharynx, ET orifice, and torus tubaris; the second pass to visualize the sphenoethmoidal recess and superior turbinate, and the third pass to visualize the middle turbinate and middle meatus. Then the patient was enquired about the pain or discomfort experienced during the procedure, and post-procedure time was noted. Then the procedure performing doctor was enquired about the excellency of visualization of the structures, areas that were difficult to visualize, and any trauma/bleeding noted during the preparation was noted. For convenience, group A has been described as the packing group and group B as the spray group.

Ethical declaration

All procedures performed in this study involving human participants were in accordance with the ethical standards of the Internal Human Ethics Committee and Scientific Research Committee of the Sri Venkateshwara Medical College Hospital and Research Centre, Puducherry, India, with reference no. SVMV/SRC/IEC/2021/20/CTR 605 and/or national research committee, the 1964 Helsinki Declaration, and its later amendments or comparable ethical standards.

Statistical analysis

All the data were analyzed through SPSS version 23 software. The Chi-square test was used, and the p-value of <0.05 was considered significant.

## Results

**Figure 1:**
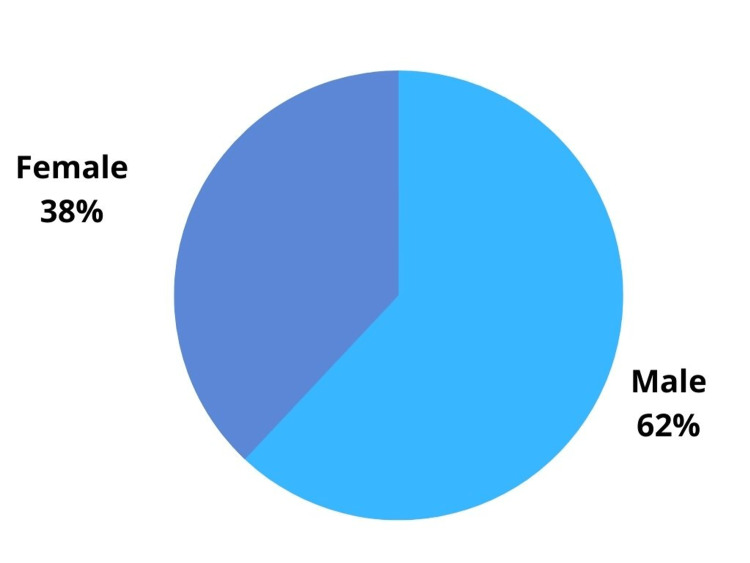
Distribution of the participants with respect to gender (n=246).

A total of 246 patients participated in the study. The mean age of the participants was 33+/-5 years. Out of 246, the majority were males, 153 (62%), and 93 (38%) females, as shown in Figure 1. Nearly 93 (75.6%) of the participants in the packing group experienced pain during packing for the procedure compared to only 30 (24.4%) patients who had pain while spraying. During the waiting period of 10 minutes for the nose preparation, 86 (69.9%) patients in the spray group experienced a burning or tingling sensation in the nose. In contrast, only 40 (32.5%) patients in the packing group noticed a similar sensation. In addition, nearly half of the patients in the packing group, 60 (48.8%), had throat pain during the waiting period in comparison to only 21 (16.3%) in the spray group with a similar feeling, as shown in Table 1.

**Table 1:** Symptoms or discomfort experienced by the patient before the endoscopy (n=246).

Category	Group A n (%)	Group B n (%)	P-value
Pain during pre-endoscopic procedure			
Present	93 (75.6)	113 (91.9)	0.0005
Absent	30 (24.4)	10 (8.1)	
Symptoms experienced during the waiting period			
Burning/tingling sensation			
Present	40 (32.5)	86 (69.9)	<0.0001
Absent	83 (67.5)	37 (30.1)	
Throat pain			
Present	60 (48.8)	21 (16.3)	<0.0001
Absent	63 (51.2)	103 (83.7)	

Patients of the packing group spent a time period of 3-10 minutes for nasal packing. Among them, about 81 (65.9%) had their packing for 5-10 minutes. However, most of the spray group participants, 116 (94.3%), had their preparation in less than a minute, as shown in Table 2. About 15 (12%) patients in the packing group had mucosal bleeding during packing in the preparation of the procedure, and only one patient experienced mucosal bleeding from the spray group. The pain was experienced during the endoscopy procedure in as high as 40 (32.5%) of the spray group participants compared to 15 (12.2%) of the packing group participants. However, 80 (65%) participants in the packing group and 32 (26.1%) in the spray group felt discomfort after the procedure, as shown in Table 3.

**Table 2:** Distribution of the participants regarding the time taken for the endoscopic procedure (n=246).

Category	Group A n (%)	Group B n (%)
Time taken for preparation		
< 1 min	0	116 (94.3)
1 to 3 minutes	0	7 (5.7)
3 to 5 minutes	42 (34.1)	0
5 to 10 minutes	81 (65.9)	0

**Table 3:** Symptoms or discomfort experienced by the patient during and post procedure (n=246).

Symptoms	Group A n (%)	Group B n (%)	P-value
Mucosal bleeding			
Present	15 (12)	1 (0.8)	0.0003
Absent	108 (88)	122 (99.2)	
Pain during the procedure			
Present	15 (12.2)	40 (32.5)	0.0001
Absent	108 (87.8)	83 (67.5)	
Discomfort after the procedure			
Present	80 (65)	32 (26.1)	<0.0001
Absent	43 (74.9)	91 (73.9)	

**Figure 2:**
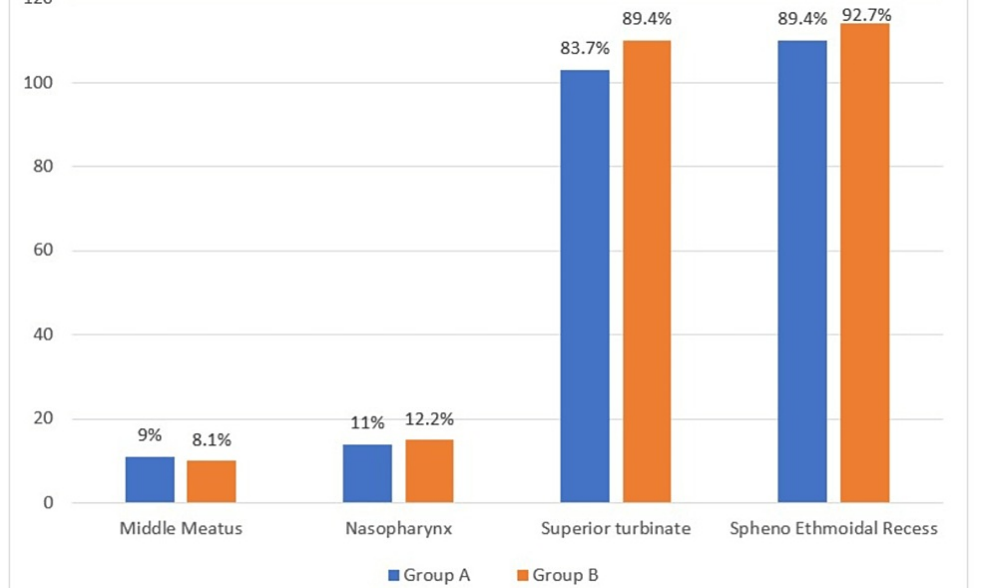
Distribution of the participants with difficulty in visualizing the structures during the endoscopic procedure (n=246).

Regarding the visualization of the nasal structures during the endoscopic procedure, no major difference in visualizing the middle turbinate and nasopharynx between the two groups was noted. Difficulty in visualizing the superior turbinate was noted in 110 (89.4%) patients from the spray group compared to 103 (83.7%) patients from the packing group. The sphenoethmoidal recess could not be visualized in 114 (92.7%) participants in the spray group and 110 (89.4%) in the packing group, as shown in Figure 2.

On assessing the association between different variables between both groups, a significant difference was noted among the pain during the pre-endoscopic procedure (p=0.0005), burning or tingling sensation, and throat pain during the waiting period (p<<0.0001), mucosal bleeding (p=0.0002), pain during the procedure (p=0.0001), and discomfort experienced after the procedure (p<0.0001).

## Discussion

This study aimed to assess the efficacy of the different modes of application of local anesthetic and decongestant on the nasal mucosa, the comforts experienced by the patient, and the doctor's easiness or ability to visualize the structures during the procedure. A total of 246 patients participated in the study, with 123 patients in each group. Overall, 93 (75.6%) participants in the packing group experienced pain during packing. Similar to our results, in a study by Mishra P et al. [9], spray group participants experienced more pain or discomfort during the preparation. However, their study used 10% lignocaine spray in the spray group, which could have produced more irritation than 4% lignocaine. To avoid bias in lignocaine concentration, we used 4% lignocaine for both the spray group and packing group. Nearly 86 (69.9%) of the spray group participants had a burning or tingling sensation in the nose after spraying the lignocaine-oxymetazoline. This is due to the fact that on spraying, the whole dose of lignocaine is delivered to the nasal cavity as a single shot, so the patient had more tingling sensation than the packing group.

In this study, about half of the participants, 60 (48.8%) of the packing group, had throat pain. Similar results were also noted in the study by Hu CT [7]. Hu CT also showed that packing group participants had more accumulation of lignocaine in the pyriform sinus, which produces throat irritation. Another important thing is that more time is taken for the pre-endoscopic preparation of the nasal cavity in the packing group than in the spray group. Mucosal bleeding was present in 15 (12%) of the participants in the packing group. In contrast, in a study by Hu CT [12] where there were 103 patients in the packing group did not have any mucosal bleeding. Mucosal bleeding was also documented in other studies, as in the study by Mishra P et al. [9], who noted mucosal bleeding from eight patients on nasal packing. Nearly 40 (32.5%) of the spray group participants experienced pain during the procedure, but only 15 (12.2%) had pain in the packing group. The higher anesthetic effect on packing can be attributed to the fact that gauze used in the nasal packing removes any dried or adherent mucoid debris or discharge, thereby increasing the absorption effect of lignocaine by mechanical cleaning effect. In addition, the contact time of anesthetic agents with the nasal mucosa is more with the packing group, which further helps reduce pain during endoscopy [13]. Other studies are available in the medical literature. Contrary to the above findings, the study by Maffei M et al. on white patients showed that spraying the nasal cavity gives faster action and is less painful compared to the packing method [14].

The results did not show any statistically significant difference in visualizing the superior turbinate and sphenoid sinus opening between the two groups. However, the packing group had less difficulty visualizing the other structures than the spray group. The main reasons for the difficulty in visualizing the structures may be due to insufficient contact time of decongestant, inadequate turbinate shrinkage, and anatomical restrictions [13-15]. Most of the patients in the packing group, 80 (65%), felt discomfort after the procedure, compared to a fewer number of 32 (26.1%) in the spray group with the same feeling. The study by Mishra P et al. showed that 8 of 50 spray group participants had some form of discomfort in the throat, and 12 of 50 patients in the packing group had heaviness and bitter taste in the throat [9]. Though the incidence is rare, events of lignocaine toxicity with anterior nasal packing have been reported. In medical literature, such events have not been identified with lignocaine spray, making it a relatively safer option [16].

## Conclusions

The DNE is a common day-to-day procedure in ENT practices. The preparation of the nose for the diagnostic endoscopy by properly decongesting and anesthetizing both nasal cavities is essential for the procedure. However, in the literature, there was not much research comparing the effects on the different modes of application of these formulations. Therefore, this study was done to compare the effects of the preparation technique during the diagnostic endoscopic procedure. The results showed that both the methods had various advantages and disadvantages, but in comparison, 4% lignocaine-oxymetazoline cotton pledgets nasal packing is found to be slightly superior to 4% lignocaine-oxymetazoline spray in decongesting and anesthetizing the nasal cavity. Since there is no significant difference in the effect on visualizing the structures of the nasal cavity, the 4% lignocaine-oxymetazoline spray can be safely used in any emergency situation and in patients who are very sensitive or apprehensive about nasal packing.
